# Supplementary material for: Pathogen-driven degradation of endogenous and therapeutic antibodies during streptococcal infections
Source: Nat Commun. 2023 Oct 23;14:6693. doi: 10.1038/s41467-023-42572-0 (PMC10593946; doi:10.1038/s41467-023-42572-0)
Supplement: Supplementary file 1 — Supplementary Information [file 41467_2023_42572_MOESM1_ESM.pdf]

EndoS cleavage products (in-vitro)

Peptide	Glycan	PSM	Protein	Species
[R].EEQYNSTYR.[V]	1xHexNAc(1)	37	IgG1	Human
[R].EEQYNSTYR.[V]	1xHexNAc(1)Fuc(1)	71	IgG1	Human
[R].EEQFNSTFR.[V]	1xHexNAc(1)	34	IgG2	Human
[R].EEQFNSTFR.[V]	1xHexNAc(1)Fuc(1)	77	IgG2	Human
[R].EEQFNSTYR.[V]	1xHexNAc(1)	12	IgG3	Human
[R].EEQFNSTYR.[V]	1xHexNAc(1)Fuc(1)	39	IgG3	Human
[R].EEQYNSTFR.[V]	1xHexNAc(1)	5	IgG4	Human
[R].EEQYNSTYR.[V]	1xHexNAc(2)Hex(3)	3	IgG1	Human
[R].EEQYNSTYR.[V]	1xHexNAc(2)Hex(4)	4	IgG1	Human
[R].EEQYNSTYR.[V]	1xHexNAc(2)Hex(5)	6	IgG1	Human
[R].EEQYNSTYR.[V]	1xHexNAc(2)Hex(6)	6	IgG1	Human
[R].EEQYNSTYR.[V]	1xHexNAc(4)Hex(3)	3	IgG1	Human
[R].EEQYNSTYR.[V]	1xHexNAc(4)Hex(3)Fuc(1)	9	IgG1	Human
[R].EEQYNSTYR.[V]	1xHexNAc(4)Hex(4)Fuc(1)	9	IgG1	Human
[R].EEQFNSTFR.[V]	1xHexNAc(2)Hex(5)	3	IgG2	Human
[R].EEQFNSTFR.[V]	1xHexNAc(2)Hex(6)	3	IgG2	Human
[R].EEQFNSTYR.[V]	1xHexNAc(2)Hex(5)	2	IgG3	Human
[R].EEQFNSTYR.[V]	1xHexNAc(3)Hex(4)	5	IgG3	Human
[R].EEQFNSTYR.[V]	1xHexNAc(4)Hex(4)Fuc(1)	2	IgG3	Human
[R].EEQFNSTYR.[V]	1xHexNAc(4)Hex(5)Fuc(1)	3	IgG3	Human
[R].EEQFNSTYR.[V]	1xHexNAc(4)Hex(5)NeuAc(1)	9	IgG3	Human
[R].EEQFNSTYR.[V]	1xHexNAc(5)Hex(4)	2	IgG3	Human
[R].EEQFNSTYR.[V]	1xHexNAc(5)Hex(5)	3	IgG3	Human
[R].EEQFNSTYR.[V]	1xHexNAc(5)Hex(5)NeuAc(1)	5	IgG3	Human
[R].EEQINSTFR.[S]	1xHexNAc(1)	29	IgG1	Mouse
[R].EEQINSTFR.[S]	1xHexNAc(1)Fuc(1)	60	IgG1	Mouse
[R].EDYNSTIR.[V]	1xHexNAc(1)	64	IgG2B	Mouse
[R].EDYNSTIR.[V]	1xHexNAc(1)Fuc(1)	99	IgG2B	Mouse
[R].EAQYNSTFR.[V]	1xHexNAc(1)	8	IgG3	Mouse
[R].EAQYNSTFR.[V]	1xHexNAc(1)Fuc(1)	25	IgG3	Mouse
[R].EEQINSTFR.[S]	1xHexNAc(2)Hex(5)	3	IgG1	Mouse
[R].EEQINSTFR.[S]	1xHexNAc(2)Hex(6)	4	IgG1	Mouse
[R].EEQINSTFR.[S]	1xHexNAc(4)Hex(3)Fuc(1)	2	IgG1	Mouse
[R].EEQINSTFR.[S]	1xHexNAc(4)Hex(4)Fuc(1)	3	IgG1	Mouse
[R].EEQINSTFR.[S]	1xHexNAc(4)Hex(5)Fuc(1)	2	IgG1	Mouse
[R].EDYNSTIR.[V]	1xHexNAc(4)Hex(5)Fuc(1)	4	IgG2B	Mouse
[R].EDYNSTIR.[V]	1xHexNAc(5)Hex(5)NeuAc(1)	2	IgG2B	Mouse

Red: Truncated structures
Green: High-mannose structures

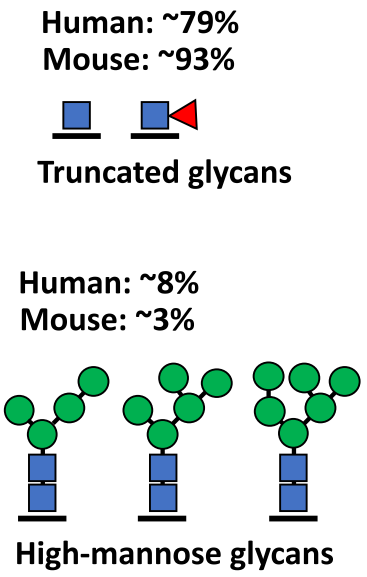

Supplemental Fig. 1. Glycoproteomics characterization of IgG products generated after exhaustive overnight incubation of human and murine plasma with recombinant EndoS. PSM: peptide spectrum matches. Hex: Hexose, HexNAc: N-acetylhexosamine, Fuc: fucose, NeuAc: N-acetylneuraminic acid, NeuGc: N-glycolylneuraminic acid.

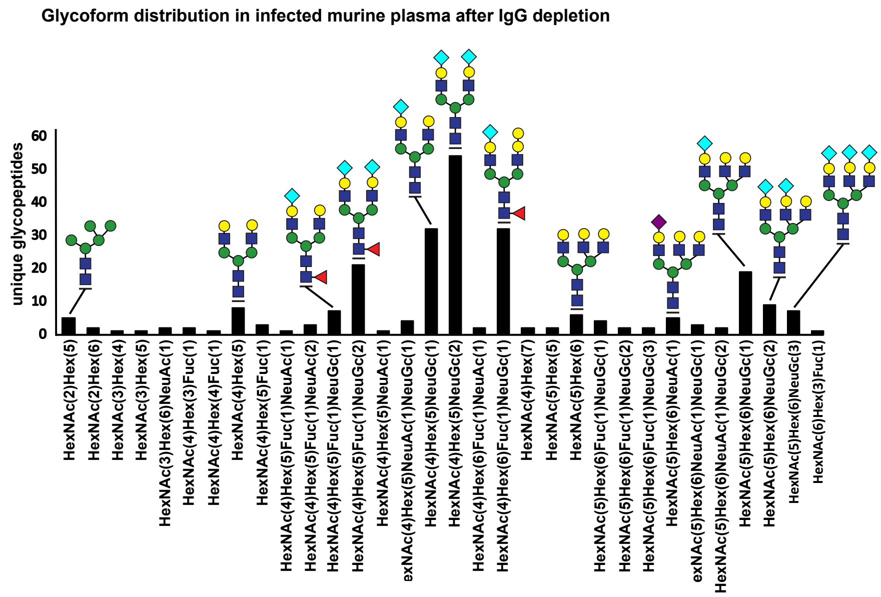

Supplemental Fig. 2. Glycoform distribution of glycopeptides derived from non-IgG glycoproteins in the plasma of GAS-infected mice at 36 p.i. Hex: Hexose, HexNAc: N-acetylglucosamine, Fuc: fucose, NeuAc: N-acetylneuraminic acid, NeuGc: N-glycolylneuraminic acid.

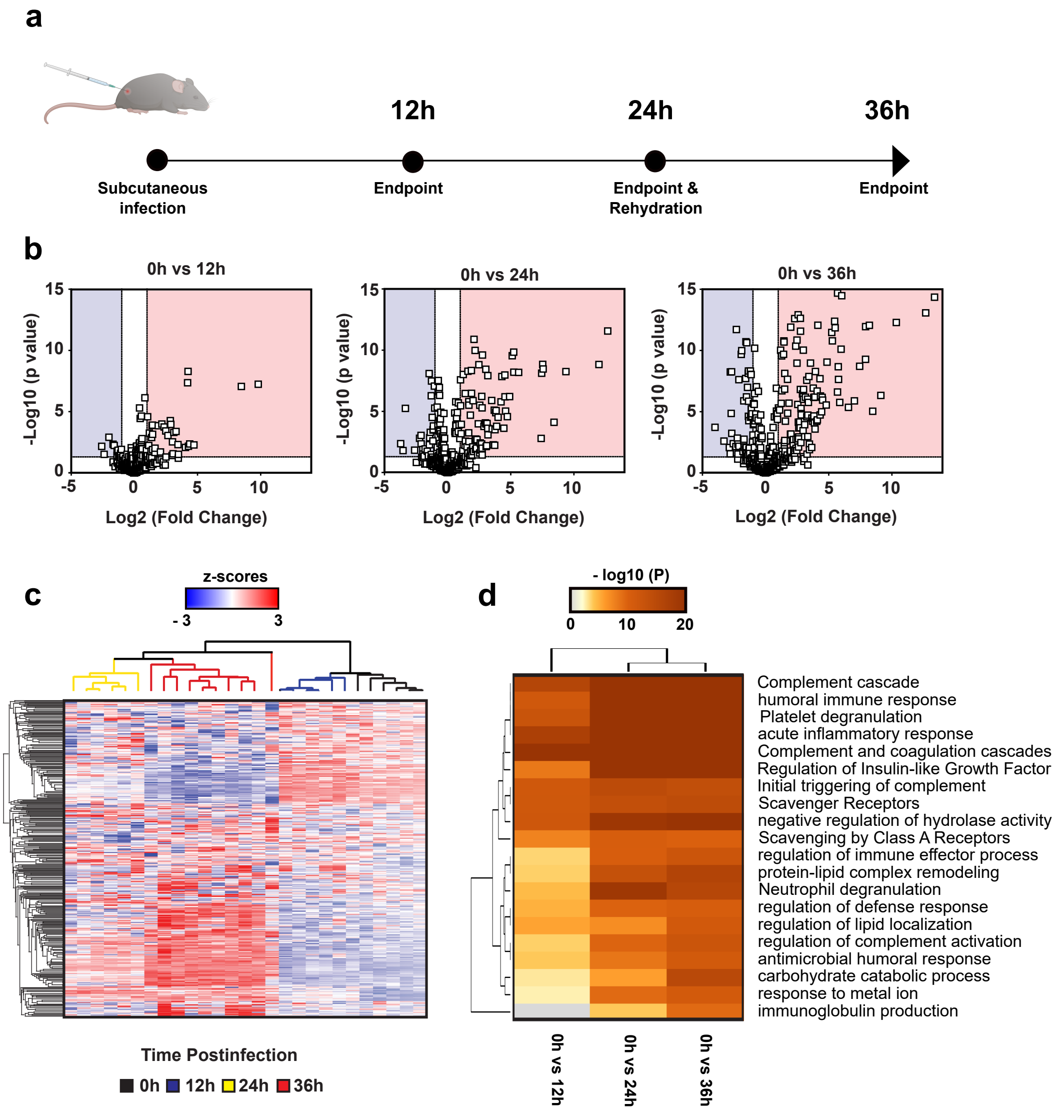

Supplemental Fig. 3. Time-resolved plasma proteome changes associated with the transition from local to systemic GAS infection. a, Schematic representation of the experimental design of the course of infection. b, Volcano plots displaying differentially changed plasma protein abundances at each time point compared to uninfected controls. c, Hierarchical clustering of plasma proteins differentially regulated over the course of the infection. d, Metascape functional enrichment analysis of c. Created with BioRender.com

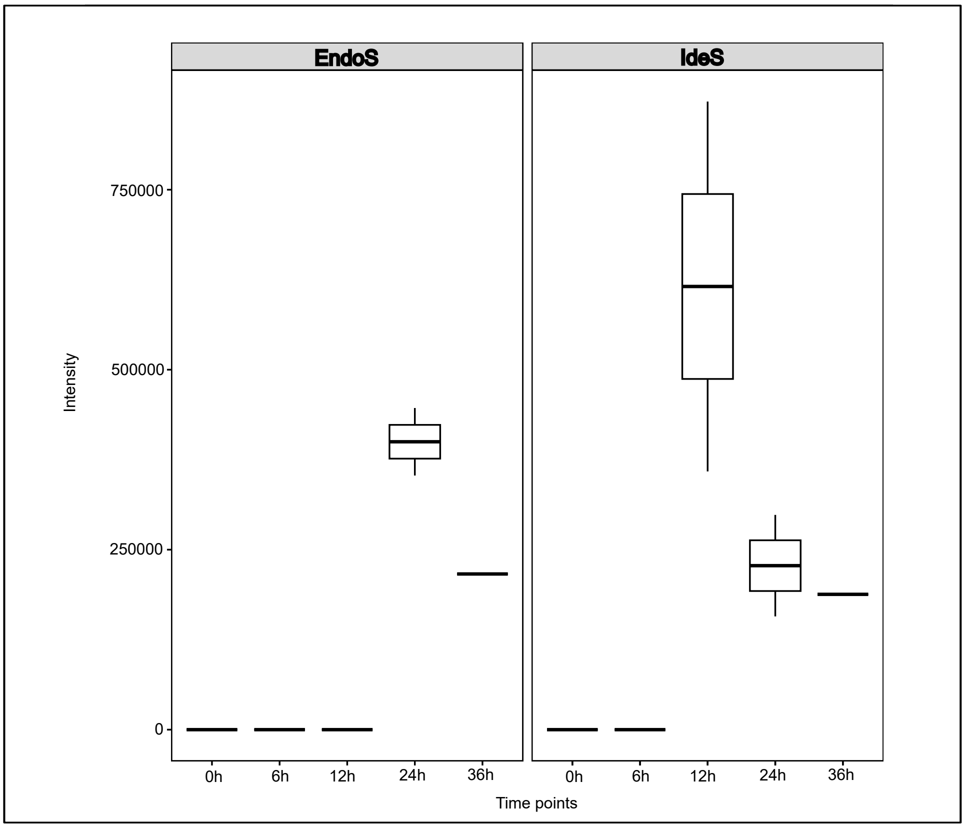

Supplemental Fig. 4. IdeS and EndoS are expressed in infected skin at the protein level. Mass spectrometry analysis of the levels of bacterial EndoS and IdeS over time in mouse skin samples from the inoculation site.

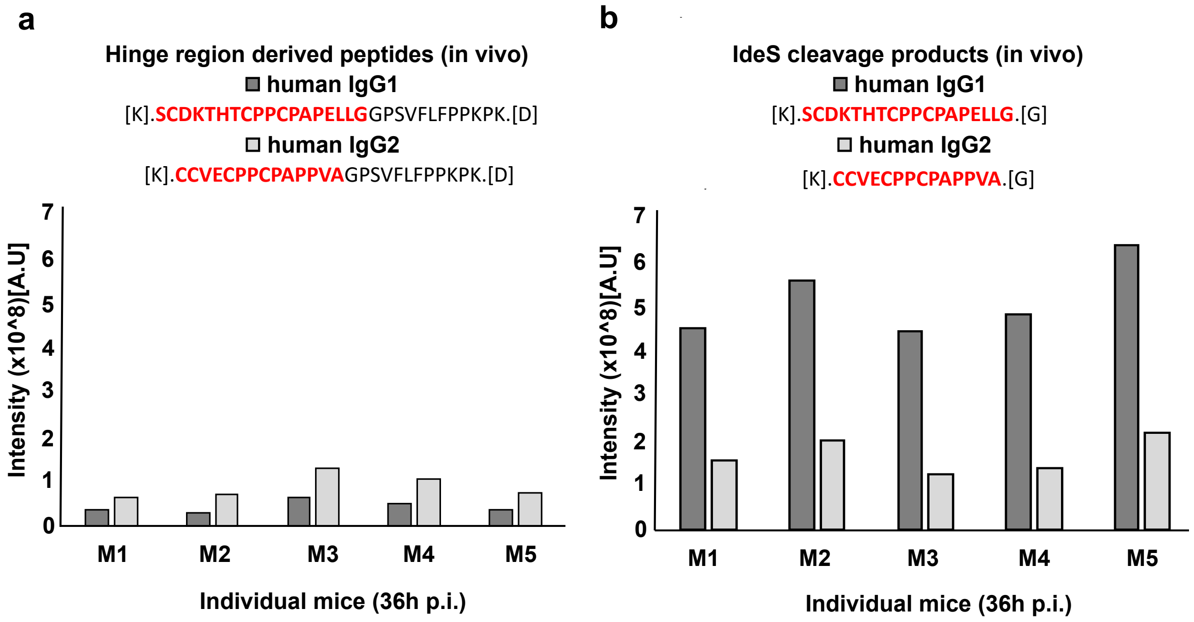

Supplemental Fig. 5. IdeS activity contributes to IVIG degradation in vivo. Mass spectrometric intensity of tryptic peptides derived from a, intact and b, IdeS-cleaved human IgG hinge regions in murine plasma upon GAS infection.

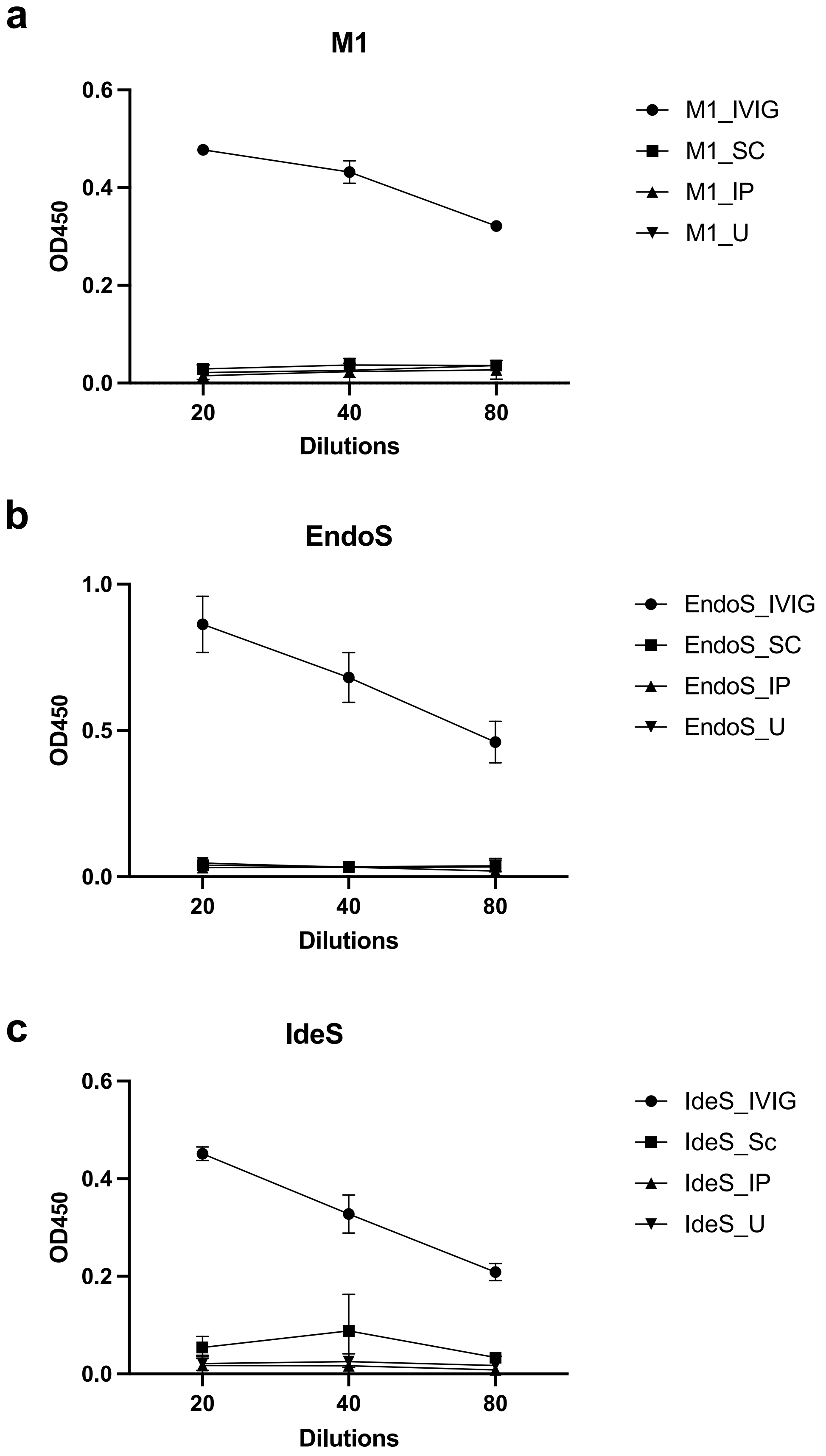

Supplemental Fig. 6. Antibody titers against a, M1, b, EndoS, and c, IdeS in IVIG and infected mouse plasma at 24 h post infection after intraperitoneal (IP) or subcutaneous (Sc) bacterial inoculation. Samples were run in triplicates, and the IgG titers for three individual mice for each infection condition are shown.

a

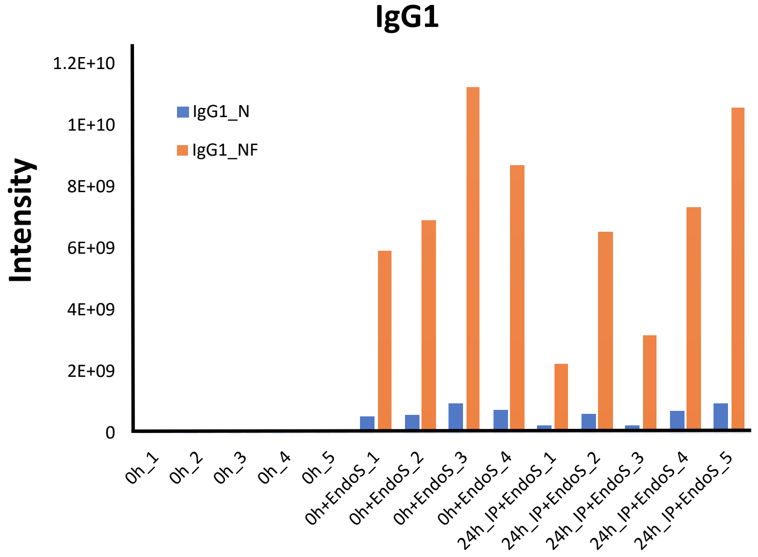

b

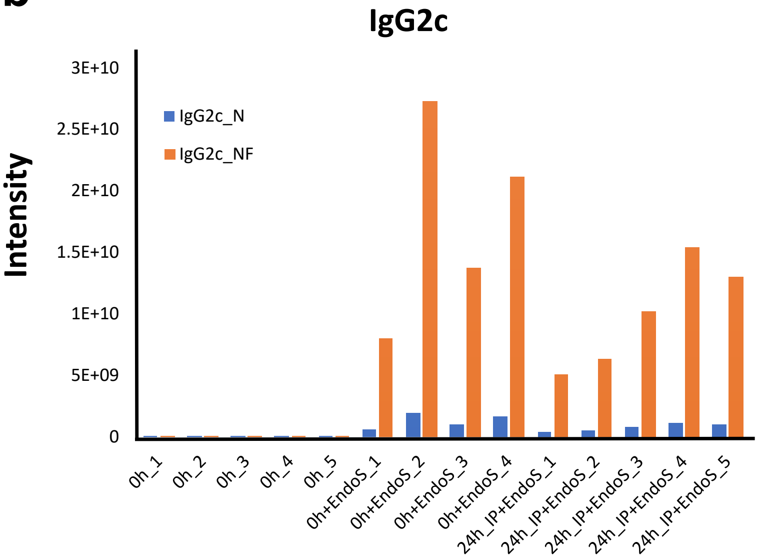

c

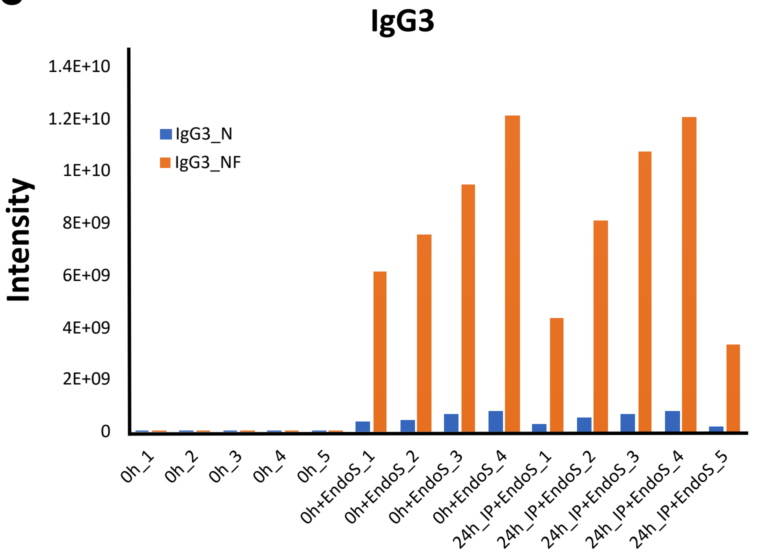

Supplemental Fig. 7. Mass spectrometric analysis of EndoS-truncated IgG glycopeptides with a single N-acetylhexosamine (N) or a single N-acetylhexosamine plus fucose (NF) derived from murine a, IgG1, b, IgG2c, and c, IgG3. Plasma from intraperitoneally infected mice at 0h and 24h post infection was incubated with or without recombinant EndoS, and the degradation products were measured by glycoproteomics. The signal for individual mice is shown in the figure. Each value of the X-axis is labeled according to the experimental conditions, 0h: uninfected, 0h+EndoS: uninfected plasma treated with EndoS, 24h_IP+EndoS: plasma from intraperitoneally (IP) infected mice at 24h post infection and treated with EndoS. The Y-axis denote the normalized peptide intensity.

Supplemental Table 1. Glycoproteomics identifications in infected murine plasma after IgG depletion

Proteins	Unique glycopeptides	Glycopeptide counts
Afamin	3	76
Alpha-1-acid glycoprotein 1	9	679
Alpha-1-acid glycoprotein 2	6	269
Alpha-1-antitrypsin 1-3	3	165
Alpha-2-macroglobulin-P	2	23
Angiotensinogen	1	5
Antithrombin-III	4	44
Apolipoprotein C-IV	2	8
Beta-2-glycoprotein 1	2	39
Carboxylesterase 1C	4	212
Carboxypeptidase B2	3	323
Carboxypeptidase N subunit 2	1	16
Ceruloplasmin	4	35
Coagulation factor V	1	2
Complement C4-B	1	2
Complement factor H	4	69
Complement factor I	2	27
Corticosteroid-binding globulin	5	43
Epidermal growth factor receptor	1	7
Fetuin-B	1	71
Fibrinogen beta chain	15	895
Fibronectin	2	84
H-2 class I histocompatibility antigen	1	103
Haptoglobin	31	1386
Hemopexin	6	246
Immunoglobulin heavy constant mu	6	119
Inter alpha-trypsin inhibitor, heavy chain 4	11	406
Inter-alpha-trypsin inhibitor heavy chain H1	2	7
Isoform 2 of Leukemia inhibitory factor receptor	4	81
Isoform D of Proteoglycan 4	1	2
Kininogen-1	6	567
Lipopolysaccharide-binding protein	1	2
Monocyte differentiation antigen CD14	2	19
Murinoglobulin-1	15	377
Phosphatidylcholine-sterol acyltransferase	1	7
Phosphatidylinositol-glycan-specific phospholipase D	5	46
Plasma kallikrein	3	18
Pregnancy zone protein	40	2901
Protein Z-dependent protease inhibitor	1	5
Prothrombin	4	145
Selenoprotein P	1	4
Serine protease inhibitor A3F	3	68
Serine protease inhibitor A3K	14	754
Serine protease inhibitor A3M	1	4
Serum amyloid P-component	2	41
Serum paraoxonase/arylesterase 1	2	32
Transthyretin	2	68
Vitronectin	5	55

Glycoproteins: 48, unique glycopeptides: 246

Supplemental Table 2. IdeS cleavage products

IdeS treatment	Identified Peptides	Protein	Species
Untreated	APELL**GG**PSVFLFPPKPK	IgG1, IgG3	Human
Untreated	CPAPELL**GG**PSVFLFPPKPK	IgG1, IgG3	Human
Untreated	ELL**GG**PSVFLFPPKPK	IgG1, IgG3	Human
Untreated	PAPELL**GG**PSVFLFPPKPK	IgG1, IgG3	Human
Untreated	PELL**GG**PSVFLFPPKPK	IgG1, IgG3	Human
Untreated	CCVECPPCPAPPV**AG**PSVFLFPPK	IgG2	Human
Untreated	CCVECPPCPAPPV**AG**PSVFLFPPKPK	IgG2	Human
Untreated	KCCVECPPCPAPPV**AG**PSVFLFPPKPK	IgG2	Human
Untreated	YGPPCPSCPAPEFL**GG**PSVF	IgG4	Human
Untreated	YGPPCPSCPAPEFL**GG**PSVFLFPPK	IgG4	Human
Untreated	YGPPCPSCPAPEFL**GG**PSVFLFPPKPK	IgG4	Human
Untreated	DCGCKPCICTVPE**VS**SVFIFPPKPK	IgG1	Mouse
Untreated	CPAPNLE**GG**PSVF	IgG2b	Mouse
Untreated	CPAPNLE**GG**PSVFIFPPNIK	IgG2b	Mouse
Untreated	CPAPNLE**GG**PSVFIFPPNIKDVLMISLTPK	IgG2b	Mouse
Untreated	IPKPSTPPGSSCPPGNIL**GG**PSVFIFPPKPK	IgG3	Mouse
Untreated	PSTPPGSSCPPGNIL**GG**PSVFIFPPKPK	IgG3	Mouse
Treated	[K].SCDKTHTCPPCPAPELL**G.[G]**	IgG1*	Human
Treated	[K].THTCPPCPAPELL**G.[G]**	IgG1*	Human
Treated	[R].KCCVECPPCPAPPV**A.[G]**	IgG2*	Human
Treated	**[L].GG**PSVFIFPPKPK.[D]	IgG3*	Mouse
Treated	**[G].G**PSVFIFPPKPK.[D]	IgG3*	Mouse
Treated	DCGCKPCICTVPE**VS**SVFIFPPKPK	IgG1	Mouse
Treated	CPAPNLE**GG**PSVF	IgG2b	Mouse
Treated	CPAPNLE**GG**PSVFIFPPNIK	IgG2b	Mouse
Treated	CPAPNLE**GG**PSVFIFPPNIKDVLMISLTPK	IgG2b	Mouse
Treated	IPKPSTPPGSSCPPGNIL**GG**PSVFIFPPKPK	IgG3	Mouse
Treated	PSTPPGSSCPPGNIL**GG**PSVFIFPPKPK	IgG3	Mouse
