# Supplementary material for: Pathogen-driven degradation of endogenous and therapeutic antibodies during streptococcal infections
Source: Nat Commun. 2023 Oct 23;14:6693. doi: 10.1038/s41467-023-42572-0 (PMC10593946; doi:10.1038/s41467-023-42572-0)
Supplement: Supplementary file 3 — Reporting Summary [file 41467_2023_42572_MOESM3_ESM.pdf]

nature portfolio

Corresponding author(s): Johan Malmström

Last updated by author(s): Sep 26, 2023

Reporting Summary

Nature Portfolio wishes to improve the reproducibility of the work that we publish. This form provides structure for consistency and transparency in reporting. For further information on Nature Portfolio policies, see our Editorial Policies and the Editorial Policy Checklist.

Statistics

For all statistical analyses, confirm that the following items are present in the figure legend, table legend, main text, or Methods section.

n/a	Confirmed	
☐	☒	The exact sample size (*n*) for each experimental group/condition, given as a discrete number and unit of measurement
☐	☒	A statement on whether measurements were taken from distinct samples or whether the same sample was measured repeatedly
☐	☒	The statistical test(s) used AND whether they are one- or two-sided<br>*Only common tests should be described solely by name; describe more complex techniques in the Methods section.*
☒	☐	A description of all covariates tested
☐	☒	A description of any assumptions or corrections, such as tests of normality and adjustment for multiple comparisons
☐	☒	A full description of the statistical parameters including central tendency (e.g. means) or other basic estimates (e.g. regression coefficient) AND variation (e.g. standard deviation) or associated estimates of uncertainty (e.g. confidence intervals)
☐	☒	For null hypothesis testing, the test statistic (e.g. *F*, *t*, *r*) with confidence intervals, effect sizes, degrees of freedom and *P* value noted<br>*Give P values as exact values whenever suitable.*
☒	☐	For Bayesian analysis, information on the choice of priors and Markov chain Monte Carlo settings
☒	☐	For hierarchical and complex designs, identification of the appropriate level for tests and full reporting of outcomes
☒	☐	Estimates of effect sizes (e.g. Cohen's *d*, Pearson's *r*), indicating how they were calculated

Our web collection on statistics for biologists contains articles on many of the points above.

Software and code

Policy information about availability of computer code

Data collection	Xcalibur (Thermo fisher, v2.2)
Data analysis	Byonic (Protein Metrics Inc, v5.0.3), Proteome Discoverer (Thermo, v.2.5.0.400), Python 3.6.10. and R version 4.3.1

For manuscripts utilizing custom algorithms or software that are central to the research but not yet described in published literature, software must be made available to editors and reviewers. We strongly encourage code deposition in a community repository (e.g. GitHub). See the Nature Portfolio guidelines for submitting code & software for further information.

Data

Policy information about availability of data

All manuscripts must include a data availability statement. This statement should provide the following information, where applicable:

- Accession codes, unique identifiers, or web links for publicly available datasets
- A description of any restrictions on data availability
- For clinical datasets or third party data, please ensure that the statement adheres to our policy

Raw mass spectrometry data associated with this manuscript is publicly available on MassIVE. Data can be found under the MassIVE ID: MSV000092131. Source data are provided with this paper.

Research involving human participants, their data, or biological material

Policy information about studies with human participants or human data. See also policy information about sex, gender (identity/presentation), and sexual orientation and race, ethnicity and racism.

Reporting on sex and gender	Not applicable
Reporting on race, ethnicity, or other socially relevant groupings	Not applicable
Population characteristics	Not applicable
Recruitment	Not applicable
Ethics oversight	Not applicable

Note that full information on the approval of the study protocol must also be provided in the manuscript.

Field-specific reporting

Please select the one below that is the best fit for your research. If you are not sure, read the appropriate sections before making your selection.

☒ Life sciences ☐ Behavioural & social sciences ☐ Ecological, evolutionary & environmental sciences

For a reference copy of the document with all sections, see nature.com/documents/nr-reporting-summary-flat.pdf

Life sciences study design

All studies must disclose on these points even when the disclosure is negative.

Sample size	The sample size was decided based on the ability of finding reproduceable proteome changes in infection models as previously assesed by us in PMID: 31604940
Data exclusions	No data was excluded
Replication	All experiments has both technical and biological replicates of at least n=3 of which all were succesfully reproduceable
Randomization	Wildtype mice were randomly allocated, same genetic background
Blinding	Was not applicable since only infected vs uninfected animals were assesed, and treatments effectes were not specifically evaluated in different disease cohorts

Reporting for specific materials, systems and methods

We require information from authors about some types of materials, experimental systems and methods used in many studies. Here, indicate whether each material, system or method listed is relevant to your study. If you are not sure if a list item applies to your research, read the appropriate section before selecting a response.

Materials & experimental systems

n/a	Involved in the study	
☐	☒	Antibodies
☐	☒	Eukaryotic cell lines
☒	☐	Palaeontology and archaeology
☐	☒	Animals and other organisms
☒	☐	Clinical data
☒	☐	Dual use research of concern
☒	☐	Plants

Methods

n/a	Involved in the study	
☒	☐	ChIP-seq
☒	☐	Flow cytometry
☒	☐	MRI-based neuroimaging

Antibodies

Antibodies used	rabbit anti-mouse IgG (1:2500, Bio-rad), (goat anti-rabbit HRP-conjugated antibody; 1:2500 Bio-Rad), anti-CD41 FITC (BD Pharmingen). Antibodies were diluted 1:200

Validation	All antibodies are commercial and have been validated by the vendors for the exact applications used in this manuscript

Eukaryotic cell lines

Policy information about cell lines and Sex and Gender in Research

Cell line source(s)	Jurkat-Lucia NFAT-CD16 and CD32 cells (invivogen)
Authentication	Cells have been authenticated bythe vendors, regarding proper morphology, growth and negative for contaminants
Mycoplasma contamination	Negative for mycoplasma
Commonly misidentified lines (See ICLAC register)	none

Animals and other research organisms

Policy information about studies involving animals; ARRIVE guidelines recommended for reporting animal research, and Sex and Gender in Research

Laboratory animals	C57BL/6 mice, Nine-weeks old
Wild animals	No wild animals were used in the study
Reporting on sex	No relevant for the study due to the small number of individuals used. Sex-based differences would require much larger sample sizes and the question was out of the scope of the study
Field-collected samples	No field collected samples were used
Ethics oversight	All animal use and procedures were approved by the local Malmö/Lund Institutional Animal Care and Use Committee, ethical permit number 03681-2019.

Note that full information on the approval of the study protocol must also be provided in the manuscript.
