# Supplementary figures and images for: Pathogen-driven degradation of endogenous and therapeutic antibodies during streptococcal infections
Source: Nat Commun. 2023 Oct 23;14:6693. doi: 10.1038/s41467-023-42572-0 (PMC10593946; doi:10.1038/s41467-023-42572-0)

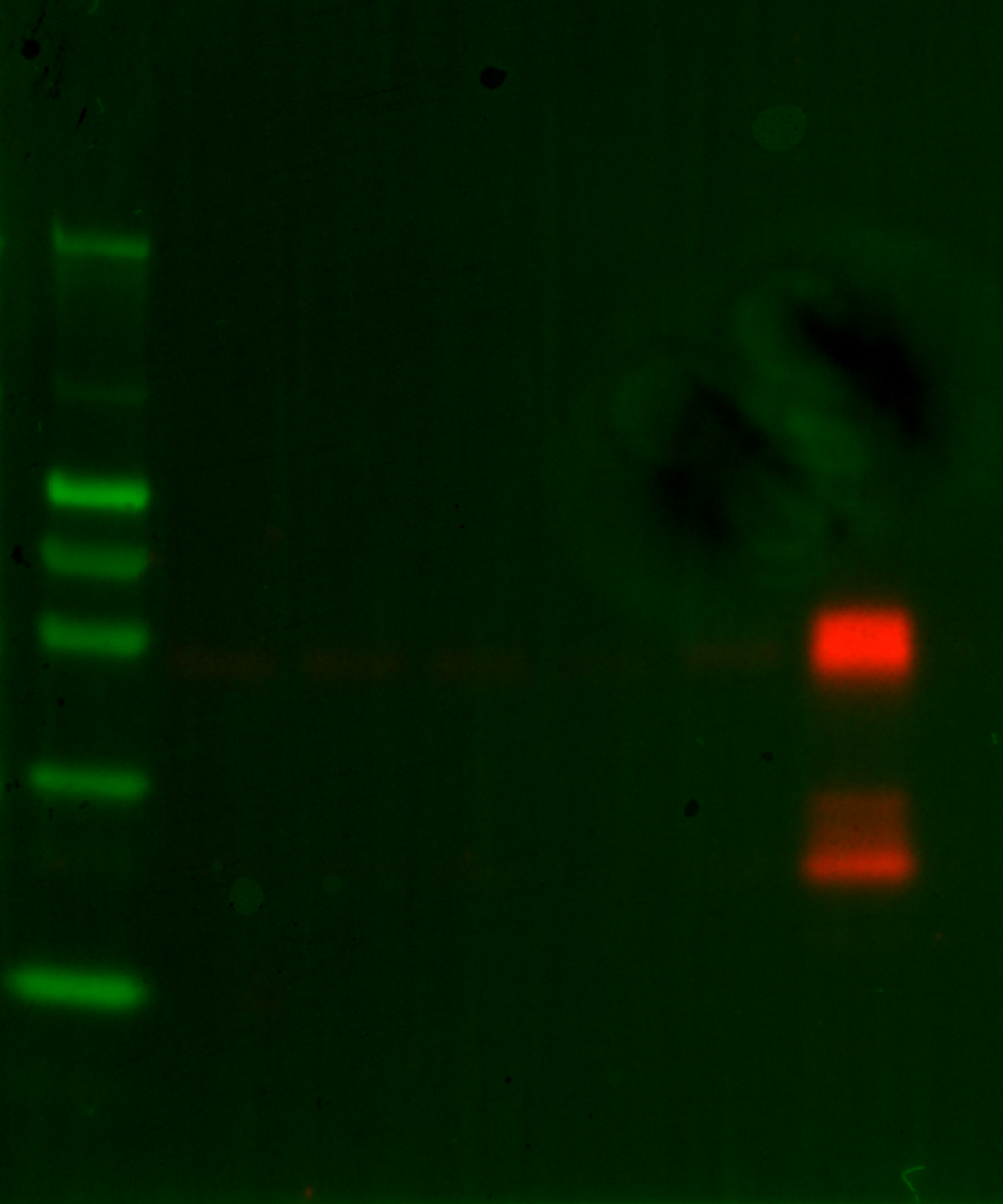

Supplement: Supplementary file 4 — Source Data [file 41467_2023_42572_MOESM4_ESM.zip › Western_Raw/2020-11-05 Kidney_anti-mouse IgG WB (Multichannel).tif]

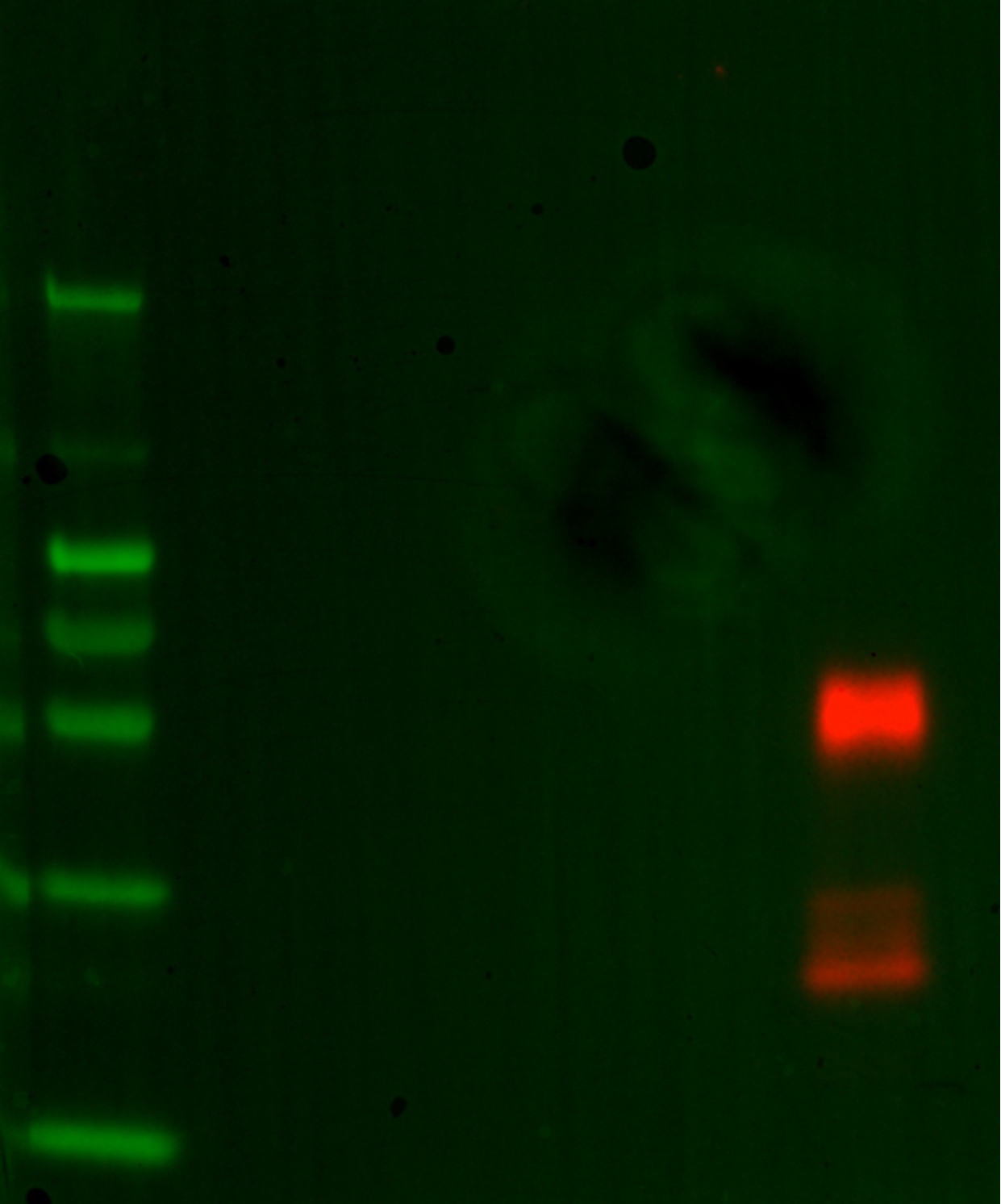

Supplement: Supplementary file 4 — Source Data [file 41467_2023_42572_MOESM4_ESM.zip › Western_Raw/2020-11-05 Liver_anti-mouse IgG WB (Multichannel).tif]

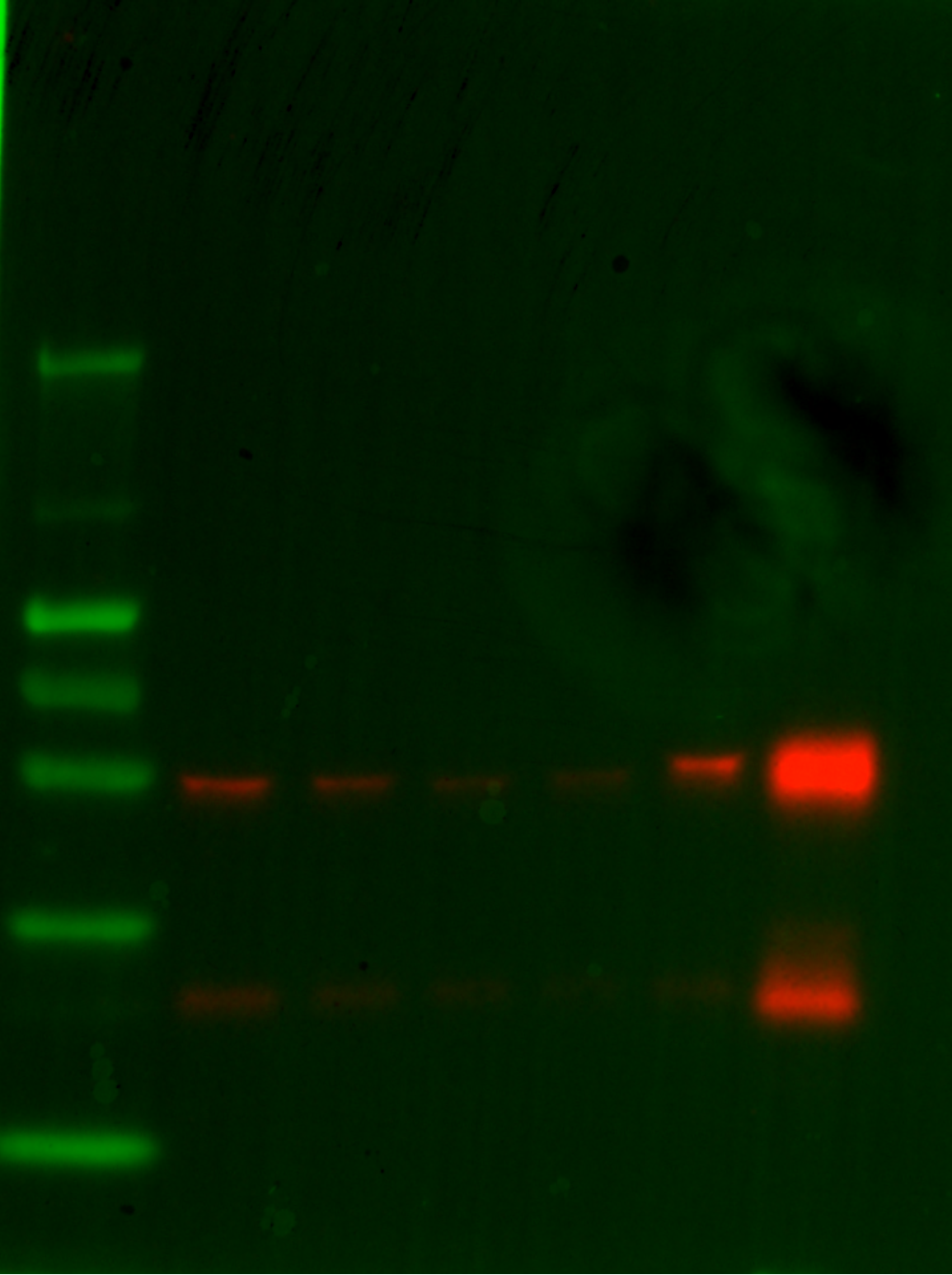

Supplement: Supplementary file 4 — Source Data [file 41467_2023_42572_MOESM4_ESM.zip › Western_Raw/2020-11-05 Plasma WB (Multichannel).tif]

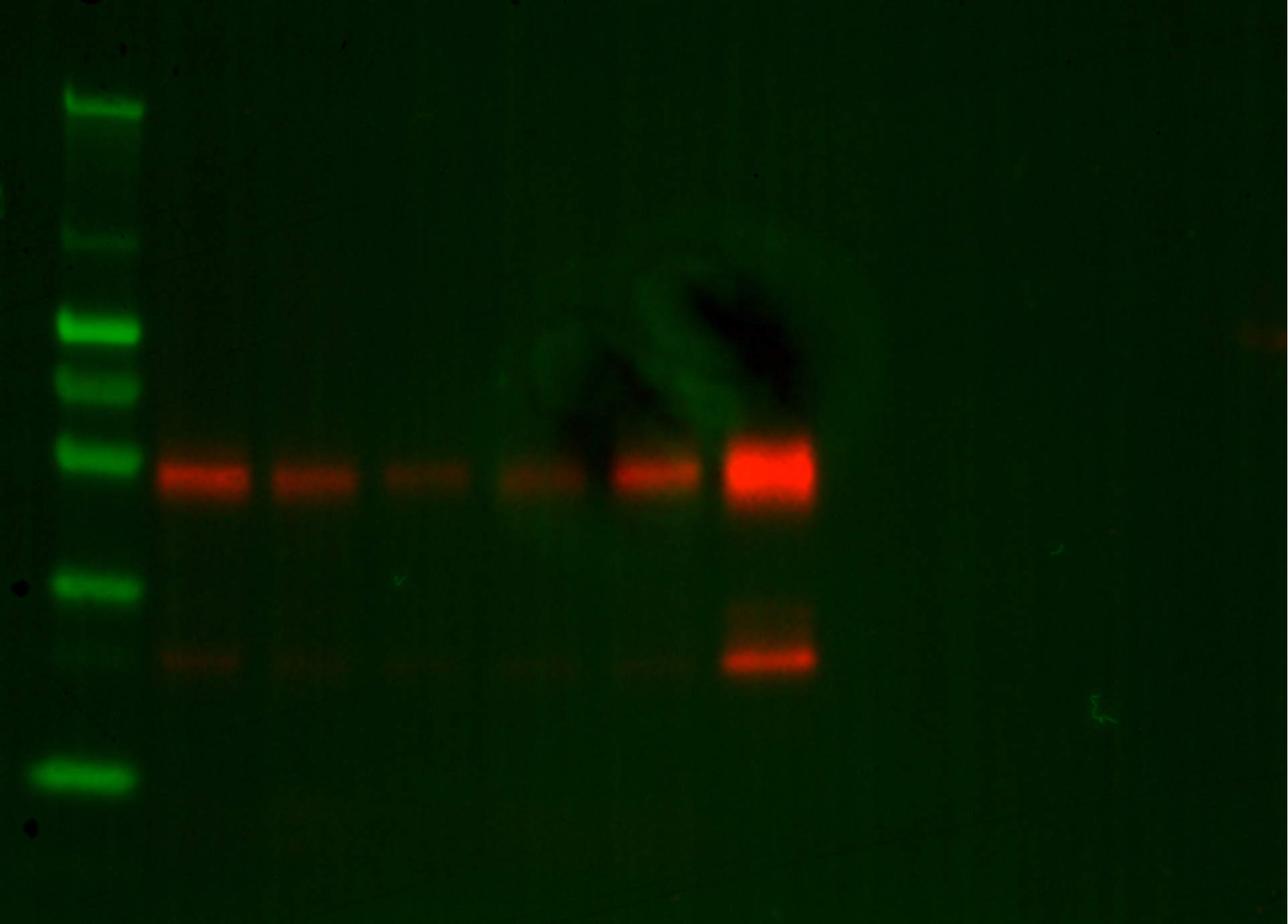

Supplement: Supplementary file 4 — Source Data [file 41467_2023_42572_MOESM4_ESM.zip › Western_Raw/20201110 Heart Anti mouse IgG WB (Multichannel).tif]

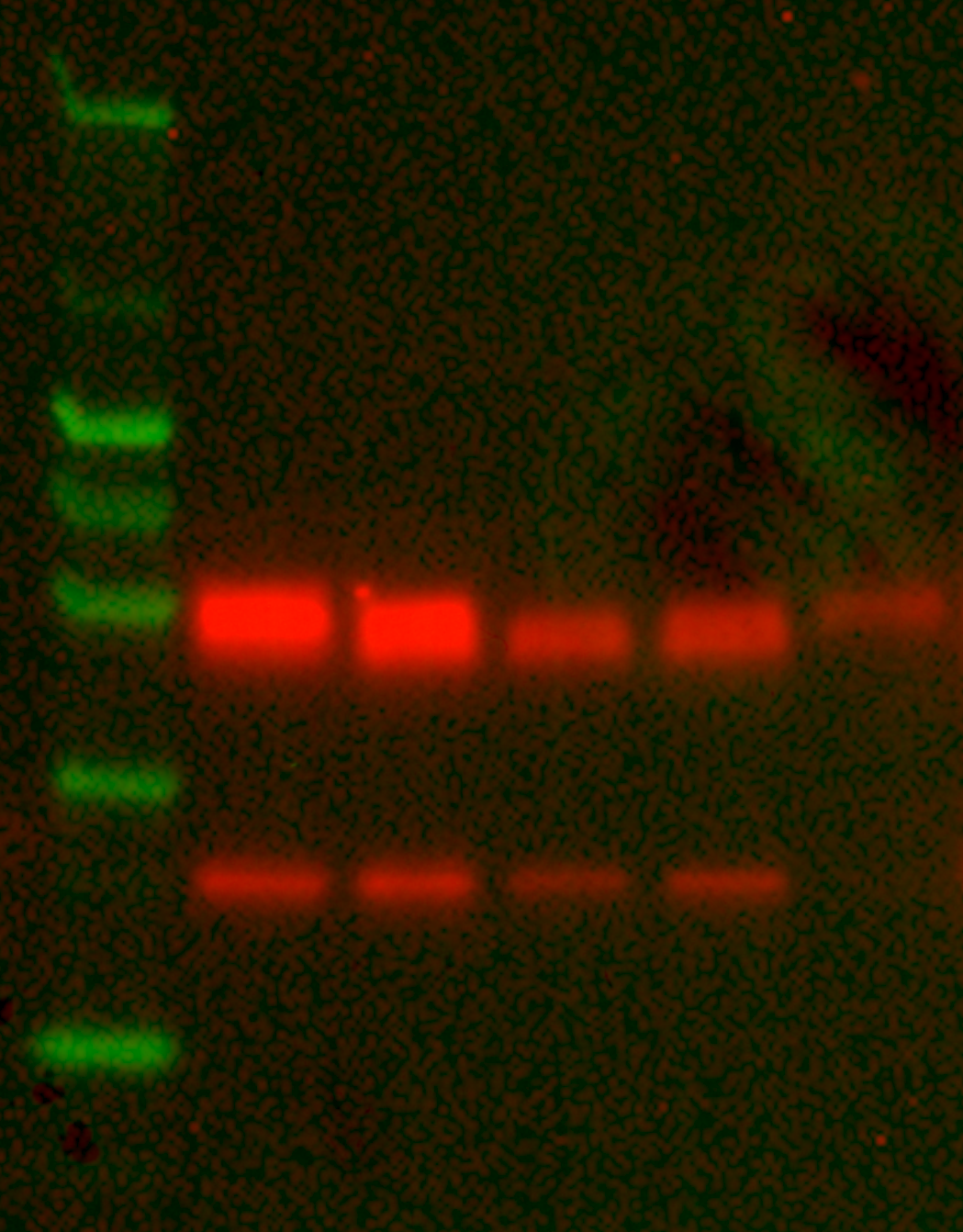

Supplement: Supplementary file 4 — Source Data [file 41467_2023_42572_MOESM4_ESM.zip › Western_Raw/2020-11-10 Skin_Anti mouse IgG WB no REFserum (Multichannel).tif]

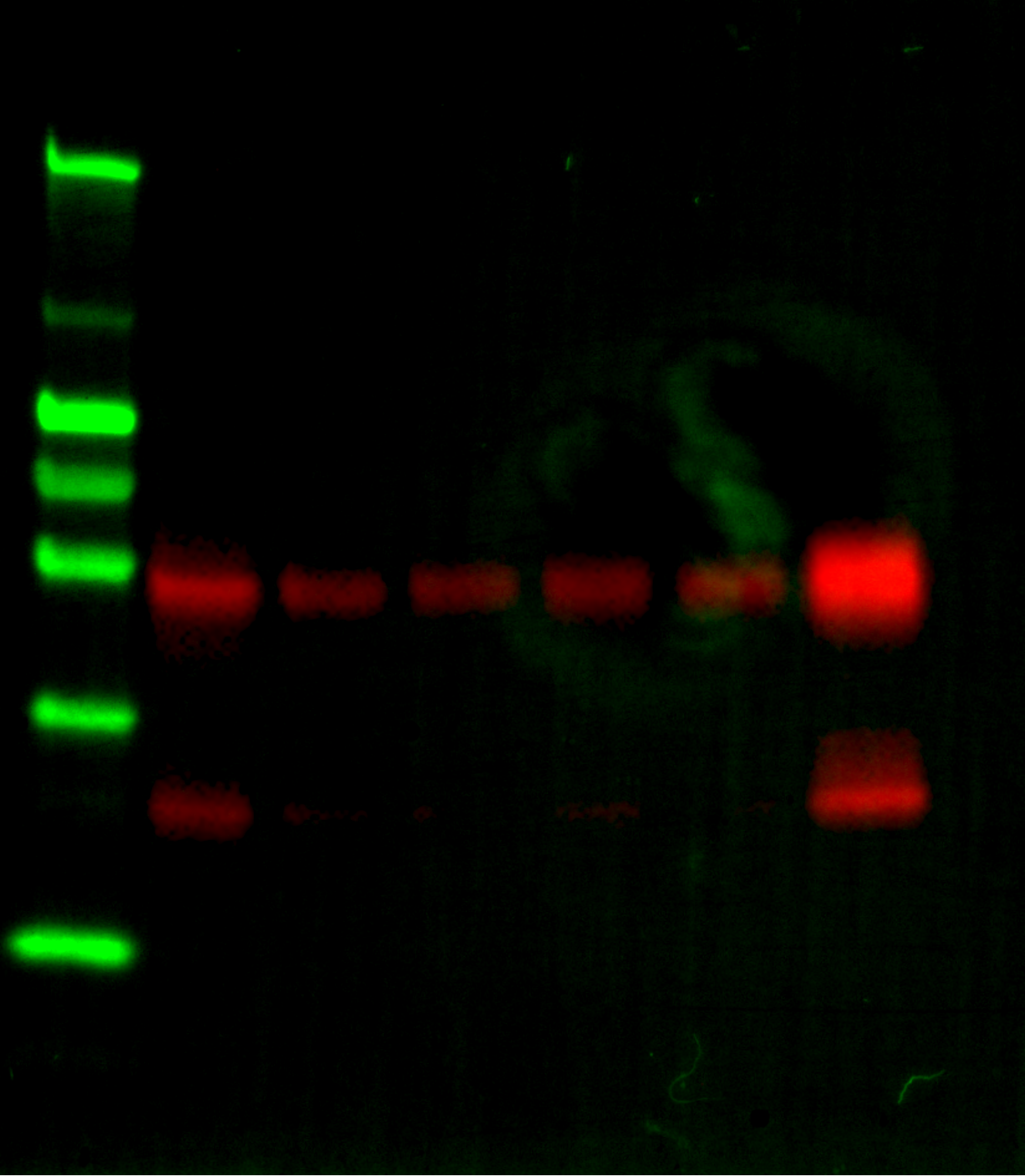

Supplement: Supplementary file 4 — Source Data [file 41467_2023_42572_MOESM4_ESM.zip › Western_Raw/2020-11-10 Spleen_Anti mouse IgG WB (Multichannel).tif]
